# Effects of Eucalyptus wood and leaf litter on saproxylic insects in the southeastern United States

**DOI:** 10.1038/s41598-024-61193-1

**Published:** 2024-05-09

**Authors:** Michael D. Ulyshen, Scott Horn, Doug Aubrey, E. Richard Hoebeke, David R. Coyle

**Affiliations:** 1 USDA Forest Service, Athens, GA USA; 2 Warnell School of Forestry and Natural Resources, University of Georgia, 180 E Green Street, Athens, GA 30602 USA; 3 Savannah River Ecology Lab, University of Georgia, PO Drawer E, Aiken, SC 29802 USA; 4 Georgia Museum of Natural History and Department of Entomology, University of Georgia, Athens, GA 30602 USA; 5 Department of Forestry and Environmental Conservation, Clemson University, Clemson, SC 29634 USA

**Keywords:** Biodiversity, Invertebrates, Novel ecosystem, Succession, Ecology, Biodiversity, Conservation biology, Forest ecology, Invasive species

## Abstract

Although *Eucalyptus* is widely planted outside its native range for timber and pulp production, the effects of these exotic plantations on biodiversity relative to native semi-natural forests or plantations of native tree species remain incompletely understood. Here, we compare the diversity of saproxylic beetles (Coleoptera) and true bugs (Hemiptera) between non-native *Eucalyptus benthamii* Maiden and Cambage (Camden white gum) and native *Pinus taeda* L. (loblolly pine) stands on the upper Coastal Plain of South Carolina, U.S.A. We sampled insects emerging from logs of both species placed in both stand types after 1, 2, 6, and 12 months in the field. Beetle and true bug richness and diversity were both significantly lower from eucalypt than from pine wood. Moreover, the two communities were compositionally distinct. Whereas pine supported many species of host-specific phloeoxylophagous beetles, most species collected from eucalypts were mycophagous or predatory taxa capable of utilizing a wide range of hosts. Species richness did not differ between logs placed in eucalypt vs. pine stands but Shannon’s diversity was significantly higher in the eucalypt stands, possibly due to greater sun exposure in the latter. Contrary to a previous study, we found no support for the idea that eucalypt litter reduces the diversity of saproxylic insects. Our findings add to the growing body of evidence that non-native plantations are less favorable to biodiversity than those consisting of native tree species.

## Introduction

As the global area of naturally-regenerating forests continues to decline, the area of planted forests, currently accounting for about 7% of global forest cover, continues to increase^1^. Plantation forests planted primarily for rapid timber production account for about 3% of current forest cover^1^. These often consist of monocultures of highly productive taxa such as *Pinus*, *Eucalyptus*, and *Populus*. These species are often planted outside their native ranges where questions arise about possible negative effects on biodiversity^2,3^. Insects dependent on dying or dead wood, termed ‘saproxylic’, are considered particularly sensitive to intensive forest management due to strong reductions in the volume and variety of dead wood^4^. Non-native plantations may exacerbate the situation by introducing novel wood species with which the local fauna has no coevolutionary history. However, the value of non-native wood species to saproxylic insects is complex and poorly understood. While some non-native species do appear to provide less preferred resources to saproxylic insects than native species^5^, especially when they are more distantly related to the native trees endemic to an area^6^, others are utilized by a comparable diversity of insects, including threatened taxa^7–11^. Such variable findings underscore the importance of assessing the suitability of non-native wood taxa to saproxylic insects on a species-by-species basis.

Saproxylic insects often exhibit a high degree of host specificity and the value of dead wood to these organisms varies greatly among both native and non-native tree taxa and even between closely related species^8^. Host specificity is especially pronounced for dying and recently dead wood when secondary metabolites and other chemical and physical properties dictate which species of phloem- and wood-feeders can colonize and survive^8^. Wood species is less important for other guilds of saproxylic insects, however. For example, generalist predators and mycophages are expected to be less impacted by wood taxa, especially as decomposition proceeds and wood becomes increasingly infiltrated by fungal tissues^6^. While this raises the possibility that non-native wood becomes more suitable to native insect communities as it decomposes, studies addressing this have reported no such pattern^5^. The rapid colonization of non-native dead wood by non-native insects such as ambrosia beetles may enhance the perceived value of this material to saproxylic insect communities at early stages of decay. Because non-native ambrosia beetles typically exhibit a broad host range, it is possible that non-native woods facilitate the proliferation of these species when introduced into new areas. However, no studies, to our knowledge, have explored this possibility.

Non-native plantation forests also have the potential to indirectly affect saproxylic insects by altering the chemistry of the forest floor through inputs of novel leaf litter. Because the litter produced by non-native plants has been shown to reduce the diversity and alter the composition of litter- and soil-dwelling arthropods^12–14^, non-native litter may reduce the suitability of dead wood for saproxylic insects. In Chile, Fierro et al.^5^ suggested that the “toxic leaf litter” produced by *Eucalyptus* contributed to the lower diversity of saproxylic beetles in remnant pine stumps and logs in eucalypt plantations compared to those in non-native pine plantations. However, because the dead wood sampled in the eucalypt and pine plantations likely differed in age (e.g., the eucalypt plantations were about half the age of the pine plantations), it is possible that such differences were unrelated to leaf litter in that study. A more controlled experiment is needed to address this question.

Exhibiting rapid growth rates and tolerating a wide range of soil conditions, commercial eucalypt plantations cover roughly 20 million hectares globally^15^, equivalent to the area of the US state of Nebraska. While *Eucalyptus* is native to Australia and some neighboring islands, over 95% of eucalypt plantations occur outside of this region, over half of which occurs in Brazil, India and China^15^. As the global area planted in eucalypt continues to grow, there is considerable interest in better understanding the ecological implications of these non-native plantations. Numerous studies have documented the deleterious effects of eucalypt plantations on a variety of native taxa—including herbaceous plants, birds, stream invertebrates, and pollinators^16–18^—compared to native forests. Although less studied, saproxylic insects are also thought to benefit little from the woody debris produced by *Eucalyptus* outside its native range^19^. However, this deserves a closer look given the high diversity of saproxylic insects associated with the genus in Australia^20,21^.

Although fast-growing native pines dominate the timber industry in the southeastern United States, there is some interest in non-native *Eucalyptus* as an even more productive alternative in places such as Florida where winters are sufficiently mild^22^. However, the ecological implications of such a decision remain largely unknown for this biodiverse region. To better understand the effects of eucalypt plantations on insect diversity, we compared the diversity and composition of saproxylic insects in non-native eucalypt vs. native pine wood at different stages of decomposition on the upper Coastal Plain of South Carolina, U.S.A. *Pinus taeda* L. was selected for this comparison because it is currently the most widely planted species across the region and is therefore the species most likely to be displaced by eucalypt plantations. We further assessed how stand type (eucalypt vs. pine), and specifically the type of litter beneath woody debris, affected wood colonization by these insects.

We hypothesized that (1) the wood of *Eucalyptus* would be utilized by fewer insect species, (2) these differences would lessen over time as decomposition progressed, and (3) the wood of both species would be colonized by a greater diversity of insects when placed in pine vs. eucalypt stands.

## Methods

**Figure 1:**
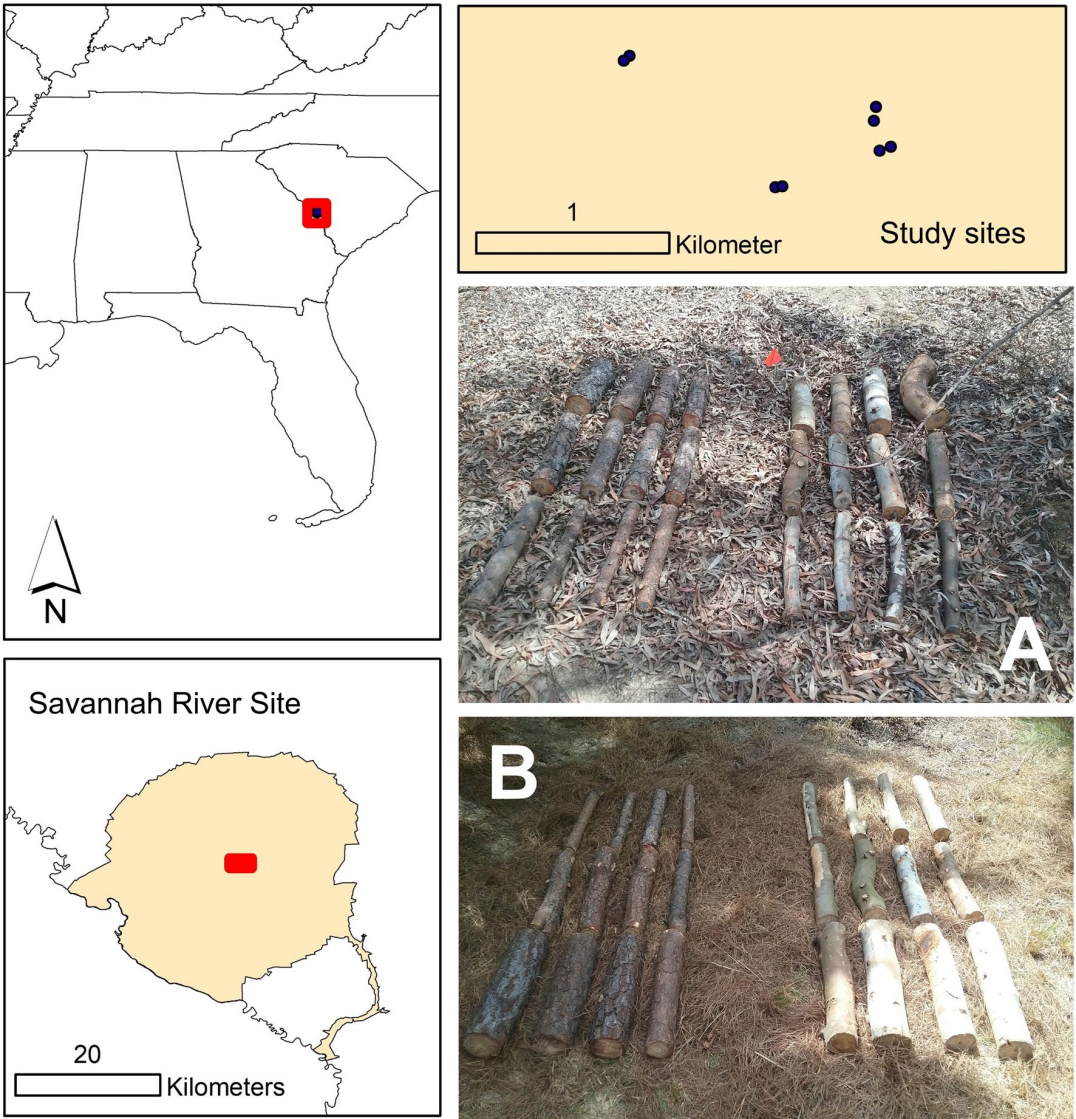
Map of study locations on the Savannah River Site in South Carolina. (**A,B**) Show the placement of logs in *Eucalyptus* and pine stands, respectively.

This study took place on the Savannah River Site, an 80,000 ha property owned by the US Department of Energy in South Carolina, USA (Fig. 1). The climate is warm temperate with an average high temperature in July of 34.4 °C, an average low temperature in January of 0.6 °C, and an average annual precipitation of 1.3 m. We utilized four locations, each consisting of an experimental *Eucalyptus benthamii* Maiden and Cambage stand within a matrix of loblolly pine. The eucalypt stands, planted in October 2013, were 0.125 ha in size, with 168 trees in 12 rows of 14 trees, whereas the surrounding loblolly pine stand was planted in January 2013 with a similar spacing and density^23,24^. The four blocks were separated from one another by about 0.1–1.5 km (Fig. 1). At each location, we established one plot within the eucalypt stand and another in the adjacent pine stand, both typically within about 10 m of the boundary between stand types. The ground surface at each eucalypt and pine plot was completely and uniformly covered by a layer of naturally senesced eucalypt and pine litter, respectively.

On 13 April 2018, 12 logs each of eucalypt and pine (cut from living trees felled for this purpose) were placed in each plot, four from each of three diameter classes: small (6.07 ± 0.14 and 6.23 ± 0.15 cm), medium (8.44 ± 0.17 and 8.49 ± 0.15 cm), and large (11.63 ± 0.27 and 11.74 ± 0.31 cm) (values are for eucalypt and pine, respectively). Despite this range in sizes, we acknowledge that this study does not capture the full range of variability in dead wood size or posture. The 12 logs from each species were grouped closely together (but not touching) in three rows by diameter class and the two species groups in each plot were separated by about 1 m (Fig. 1). We returned to collect one log of each diameter class from each species after 1, 2, 6, and 12 months in the field. To prevent the movement of insects between logs from different species or plots during transport, each set of three logs was enclosed within a sealed plastic bag. Each set was then bundled together and placed within an aerated rearing bag to collect emerging insects over a period of 12 months following the methods described by Ulyshen and Hanula^25^. All captured beetles (Coleoptera) and true bugs (Hemiptera) were pinned and identified by MU and ERH to the lowest taxonomic unit possible, typically to species^26–29^.

Unless otherwise stated, all analyses were conducted in R^30^. We calculated the total richness and Shannon’s diversity of beetles and true bugs by block, stand, and month of collection. We also calculated these metrics after pooling across sampling periods. These were the response variables compared among treatments using generalized linear mixed effects models with block (i.e., location) treated as the random term. Initially, stand, wood species, and their interaction were included in the models as fixed effects, but the interaction term was insignificant in all models and was therefore dropped for the final analysis. We used the Poisson and Gaussian distributions for models of richness and diversity, respectively. Finally, to test for compositional differences in communities between wood species, we conducted non-metric multidimensional scaling (NMDS) followed by PERMANOVA in PC-ORD^31^. We ran this analysis for each sampling period separately and also after pooling data across sampling periods.

### Collection and use of plant materials

Plants were collected and used on public lands with permission and in accordance with all the relevant guidelines.

## Results

**Figure 2:**
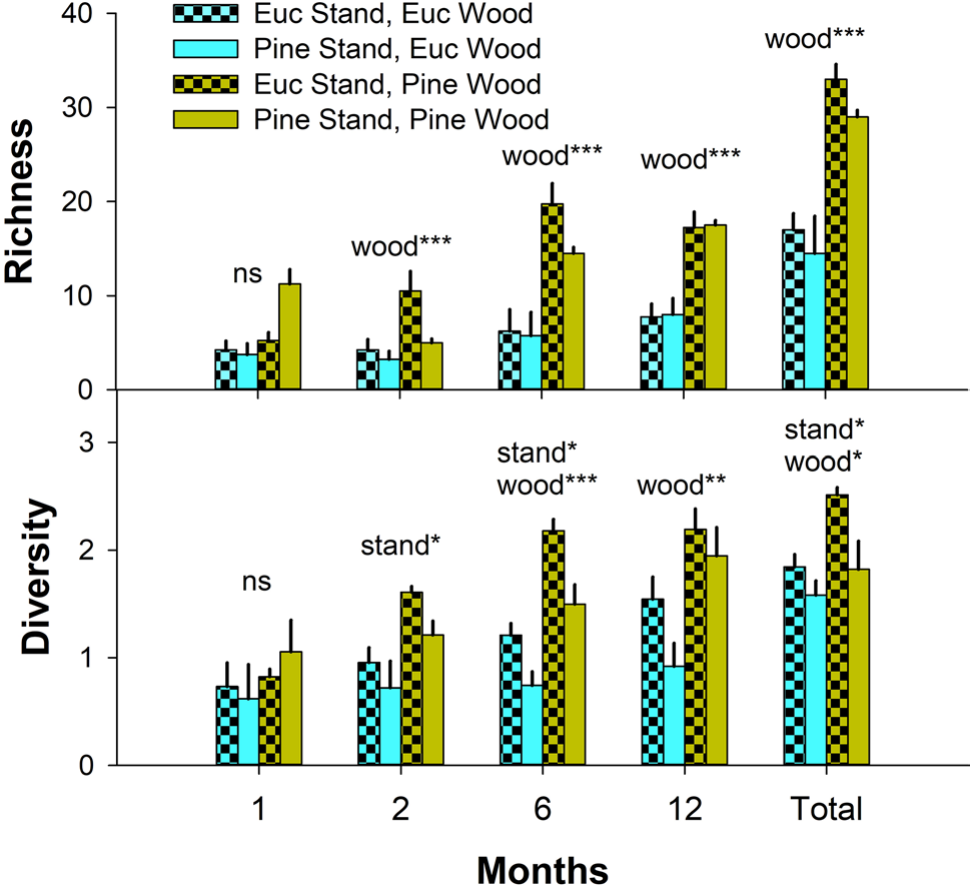
Mean ± SE beetle richness (top) and Shannon’s diversity (bottom) collected from *Eucalyptus* and pine wood placed in both stand types (*Eucalyptus* and pine). Results are shown for collections after 1, 2, 6, and 12 months in the field as well as in total, after summing across collection periods. Asterisks denote significance as follows: ***p < 0.001, **p < 0.01, *p < 0.05.

A total of 11,440 beetles from at least 95 taxa emerged from all logs over the course of the study (Table 1). Eucalypt logs produced 2674 beetles from 51 taxa while those of pine yielded 8766 beetles from 73 taxa. Overall, about twice as many beetle species on average were collected from pine compared to eucalypt, a significant difference (Table 2, Fig. 2). This difference was consistent across collection periods except for the 1-month sample for which there was no significant difference between wood species (Table 2). Beetle diversity was also significantly higher from pine overall and for the 6-month and 12-month collection periods (Table 2). Additionally, independent of species, beetle diversity was significantly higher from logs placed in eucalypt stands than from those placed in pine stands. This was true overall and for the 2-month and 6-month samples (Table 2).

**Table 1:** List of Coleoptera and Hemiptera species that emerged from *Eucalyptus* and pine after 1, 2, 6, and 12 months in South Carolina, USA.

Order	Family	Species	Larval guild	*Eucalyptus*				*Pinus taeda*				Total
				1 mo.	2 mo.	6 mo.	12 mo.	1 mo.	2 mo.	6 mo.	12 mo.	
Coleoptera	Aderidae	*Cnopus impressus* (LeConte)	m?	0	0	0	0	1	1	2	0	4
		*Ganascus ventricosus* (LeConte)	m?	0	0	0	3	0	0	45	21	69
		*Zonantes hubbardi* Casey	m?	0	0	0	1	0	0	0	0	1
	Biphyllidae	*Diplocoelus rudis* (LeConte)	m?	2	23	57	53	0	31	52	45	263
	Bostrichidae	*Stephanopachys rugosus* (Olivier)	px	0	0	0	0	13	5	0	0	18
	Buprestidae	*Acmaeodera tubulus* (Fabricius)	px	0	1	0	0	0	0	0	1	2
		*Buprestis lineata* Fabricius	px	0	0	0	0	8	61	47	14	130
		*Buprestis maculipennis* Gory	px	0	0	0	0	0	16	17	2	35
		*Chrysobothris cribraria* Mannerheim	px	0	0	0	0	7	9	11	1	28
		*Chrysobothris dentipes* (Germar)	px	0	0	0	0	1	5	5	0	11
		*Dicerca punctulata* (Schönherr)	px	0	0	0	0	11	4	10	3	28
	Carabidae	*Mioptachys flavicauda* (Say)	p	0	0	0	0	0	0	0	13	13
		*Tachyta nana* (Say)	p	0	0	0	0	0	0	4	17	21
	Cerambycidae	*Acanthocinus obsoletus* (Olivier)	px	0	0	0	0	0	0	1	0	1
		*Arhopalus rusticus* (L.)	px	0	0	0	0	0	1	0	1	2
		*Asemum striatum* (L.)	px	0	0	0	0	22	0	0	0	22
		*Astylopsis sexguttata* (Say)	px	0	0	0	0	0	24	31	14	69
		*Eupogonius tomentosus* (Haldeman)	px	0	0	0	0	3	0	1	3	7
		*Knulliana cincta* (Drury)	px	7	4	0	1	0	0	0	0	12
		*Monochamus caroliniensis* (Olivier)	px	0	0	0	0	10	37	13	9	69
		*Typocerus zebra* (Olivier)	px	0	0	0	0	0	1	1	0	2
	Cerylonidae	*Philothermus glabriculus* LeConte	m?	0	0	0	27	0	0	5	39	71
	Chrysomelidae	Bruchinae sp.	na	0	1	0	0	0	0	0	0	1
		*Donacia* sp.	na	0	0	1	0	0	0	0	0	1
	Ciidae	*Cis miles* (Casey)	m	0	0	0	5	0	0	0	0	5
		*Cis rotundulus* Lawrence	m	0	0	51	9	2	9	804	1109	1984
		*Cis ursulinus* Casey	m	0	0	0	0	0	0	3	3	6
	Corylophidae	Corylophidae sp. 1	m	3	0	0	0	0	0	0	0	3
		Corylophidae sp. 2	m	37	0	0	0	0	0	0	0	37
	Curculionidae	*Acalles porosus* Blatchley	px?	0	0	0	8	0	0	0	0	8
		*Ambrosiodmus rubricollis* (Eichhoff)*	m	2	0	0	0	0	3	0	0	5
		*Cossonus* sp.	m?	0	1	0	0	0	102	110	37	250
		*Cyclorhipidion bodoanum* (Reitter)*	m	0	2	0	0	0	0	0	0	2
		*Dryoxylon onoharaense* (Murayama)*	m	0	21	0	0	0	0	0	0	21
		*Hylastes tenuis* Eichhoff	px	0	0	0	0	19	1	0	0	20
		*Hypothenemus crudiae* (Panzer)	px	0	25	0	0	0	0	0	0	25
		*Hypothenemus* sp.	px	0	41	0	0	0	0	0	0	41
		*Ips calligraphus* (Germar)	px	0	0	0	0	56	2	0	0	58
		*Ips grandicollis* (Eichhoff)	px	1	0	0	0	236	18	0	0	255
		*Monarthrum mali* (Fitch)	m	472	0	0	0	0	0	0	0	472
		*Orthotomicus caelatus* (Eichhoff)	px	0	0	0	0	71	93	41	0	205
		*Pachylobius picivorus* (Germar)	px	0	0	0	0	0	1	0	0	1
		*Pissodes nemorensis* Germar	px	0	0	0	0	1	1	0	0	2
		*Pityophthorus* sp.	px	0	0	0	0	0	18	0	0	18
		*Xyleborinus saxesenii* Ratzeburg*	m	0	184	0	0	0	34	0	0	218
		*Xyleborus pubescens* Zimmermann	m	0	0	0	0	14	9	0	0	23
		*Xylosandrus crassiusculus* (Motschulsky)*	m	17	0	0	0	0	0	0	0	17
	Elateridae	*Athous cucullatus* (Say)	p	0	0	0	0	0	0	0	1	1
		*Dipropus soleatus* (Say)	p	0	0	0	0	0	0	1	0	1
		*Lacon impressicollis* (Say)	p	0	0	0	0	0	0	2	1	3
	Histeridae	*Bacanius punctiformis* (LeConte)	p	0	0	0	46	0	0	1	27	74
	Laemophloeidae	*Cryptolestes* sp.	m	6	45	14	46	0	53	17	1	182
		*Lathropus vernalis* LeConte	m?	0	2	0	0	0	1	0	0	3
	Melandryidae	*Microtonus sericans* LeConte	m?	0	0	0	0	0	0	0	1	1
	Mordellidae	*Conalia helva* (LeConte)	m?	0	0	0	0	0	0	1	2	3
		*Mordella atrata* Melsheimer	m?	0	0	0	0	0	0	1	0	1
		*Mordellaria borealis* (LeConte)	m?	0	0	0	0	0	0	2	4	6
		*Mordellistena masoni* Liljeblad	m?	0	0	0	0	1	0	0	0	1
	Mycetophagidae	*Litargus* sp.	m?	1	0	0	0	0	0	0	0	1
	Scarabaeidae	*Ataenius imbricatus* (Melsheimer)	?	0	0	1	0	0	0	0	0	1
		*Ataenius* sp.	?	0	0	1	0	0	0	0	1	2
	Silvanidae	*Ahasverus rectus* LeConte	m?	140	119	2	0	12	219	1	0	493
		*Silvanus muticus* Sharp	m?	333	188	1	1	125	0	0	0	648
	Staphylinidae	Aleocharinae sp. 1	?	0	0	0	0	0	0	0	5	5
		Aleocharinae sp. 2	?	1	1	0	0	0	0	0	1	3
		*Anacyptus testaceus* (LeConte)	i	0	0	0	0	0	0	1	83	84
		*Echiaster* sp.	?	0	0	0	2	0	0	0	0	2
		*Euconnus* sp.	?	0	0	0	1	0	0	2	4	7
		*Eumicrota* sp.	?	0	0	0	0	0	0	0	3	3
		*Leptusa* sp. nr cribratula	?	0	0	3	5	0	0	49	80	137
		*Mycetoporus floridensis* Campbell ?	?	0	0	0	1	0	0	0	0	1
		*Pycnoglypta fracta* (Casey)	?	0	0	0	2	0	0	0	0	2
		*Scaphidium* sp.	m	0	0	0	0	0	0	0	1	1
		*Scaphisoma* sp.	m	0	0	0	0	0	0	0	2	2
		*Sepedophilus* sp. 2	m	0	0	0	3	0	0	0	0	3
		*Sepedophilus* sp. 3	m	0	0	0	1	0	0	0	9	10
		*Sepedophilus* sp. cf *basalis*	m	0	0	0	2	0	1	1	16	20
		*Sunius debilicornis* (Wollaston)	?	0	8	0	4	0	0	0	0	12
		*Thoracophorus costalis* (Erichson)	m	0	16	10	492	0	1164	52	1277	3011
		*Trichopsenius* sp.	i	0	0	0	1	0	0	0	3	4
	Tenebrionidae	*Platydema flavipes* (Fabricius)	m	0	0	2	3	0	15	92	77	189
		*Polypleurus perforatus* (Germar)	m?	0	0	0	0	0	0	0	5	5
	Trogossitidae	*Temnochila virescens* (Fabricius)	p	0	0	0	0	3	0	0	0	3
		*Tenebroides* sp.	p	4	0	0	0	0	1	0	0	5
	Zopheridae	*Bitoma quadriguttata* (Say)	m?	11	3	0	1	0	0	8	3	26
		*Colydium lineola* Say	p	2	0	0	0	0	0	0	0	2
		*Endeitoma dentata* (Horn)	m?	0	2	1	5	0	960	111	82	1161
		*Hyporhagus punctulatus* Thomson	m	0	0	0	0	1	1	0	0	2
		*Namunaria guttulata* (LeConte)	m?	0	0	0	0	0	0	1	0	1
		*Paha laticollis* (LeConte)	m?	1	0	0	5	0	0	9	14	29
		*Pycnomerus haematodes* (Fabricius)	m?	0	0	0	0	0	0	0	1	1
		*Pycnomerus sulcicollis* LeConte	m?	0	0	4	70	0	71	289	178	612
Hemiptera	Achilidae	*Catonia* sp. cf *pini*	m?	0	0	0	1	0	0	6	15	22
		*Cixidia fusca* (Walker)	m?	0	0	0	0	0	0	62	7	69
	Aradidae	*Quilnus niger* (Stål)	m?	0	0	0	0	0	0	21	8	29
		Total individuals		1040	687	148	799	617	2972	1933	3244	11,440
		Total species		17	19	13	28	21	34	41	47	95

The names of exotic ambrosia beetles are followed by asterisks. Approximate guild designations are as follows: *p* predator, *m*  mycophage, *px*  phloeoxylophage, *i*  inquiline. Guilds are only assigned to species known or suspected to be saproxylic, otherwise species are marked as “na”.

**Table 2:** Results from generalized linear mixed models comparing saproxylic insect richness and diversity between Eucalyptus and pine stands and between Eucalyptus and pine wood at four points in time (1, 2, 6, and 12 months) and after pooling across all sample periods.

	Months	Stand type		Wood species	
		Estimate ± SE	Statistic	Estimate ± SE	Statistic
Richness	1	0.12 ± 0.22	z = 0.56, p = 0.58	0.02 ± 0.22	z = 0.11, p = 0.91
	2	− 0.11 ± 0.18	z = − 0.62, p = 0.53	0.78 ± 0.19	z = 4.07, p < 0.01
	6	− 0.19 ± 0.16	z = − 1.20, p = 0.23	1.44 ± 0.2	z = 7.11, p < 0.01
	12	− 0.08 ± 0.14	z = − 0.55, p = 0.58	0.86 ± 0.15	z = 5.73, p < 0.01
	Total	− 0.14 ± 0.1	z = − 1.3, p = 0.18	0.68 ± 0.11	z = 6.19, p < 0.01
Diversity	1	0.2 ± 0.14	t = 1.39, p = 0.19	0.28 ± 0.14	z = 1.96, p = 0.08
	2	− 0.57 ± 0.22	z = − 2.65, p = 0.02	0.42 ± 0.22	z = 1.94, p = 0.07
	6	− 0.47 ± 0.20	z = − 2.37, p = 0.03	1.01 ± 0.19	z = 5.12, p < 0.01
	12	− 0.43 ± 0.20	z = − 2.09, p = 0.06	0.83 ± 0.20	z = 4.06, p < 0.01
	Total	− 0.48 ± 0.17	z = − 2.87, p = 0.01	0.46 ± 0.17	z = 2.74, p = 0.02

**Figure 3:**
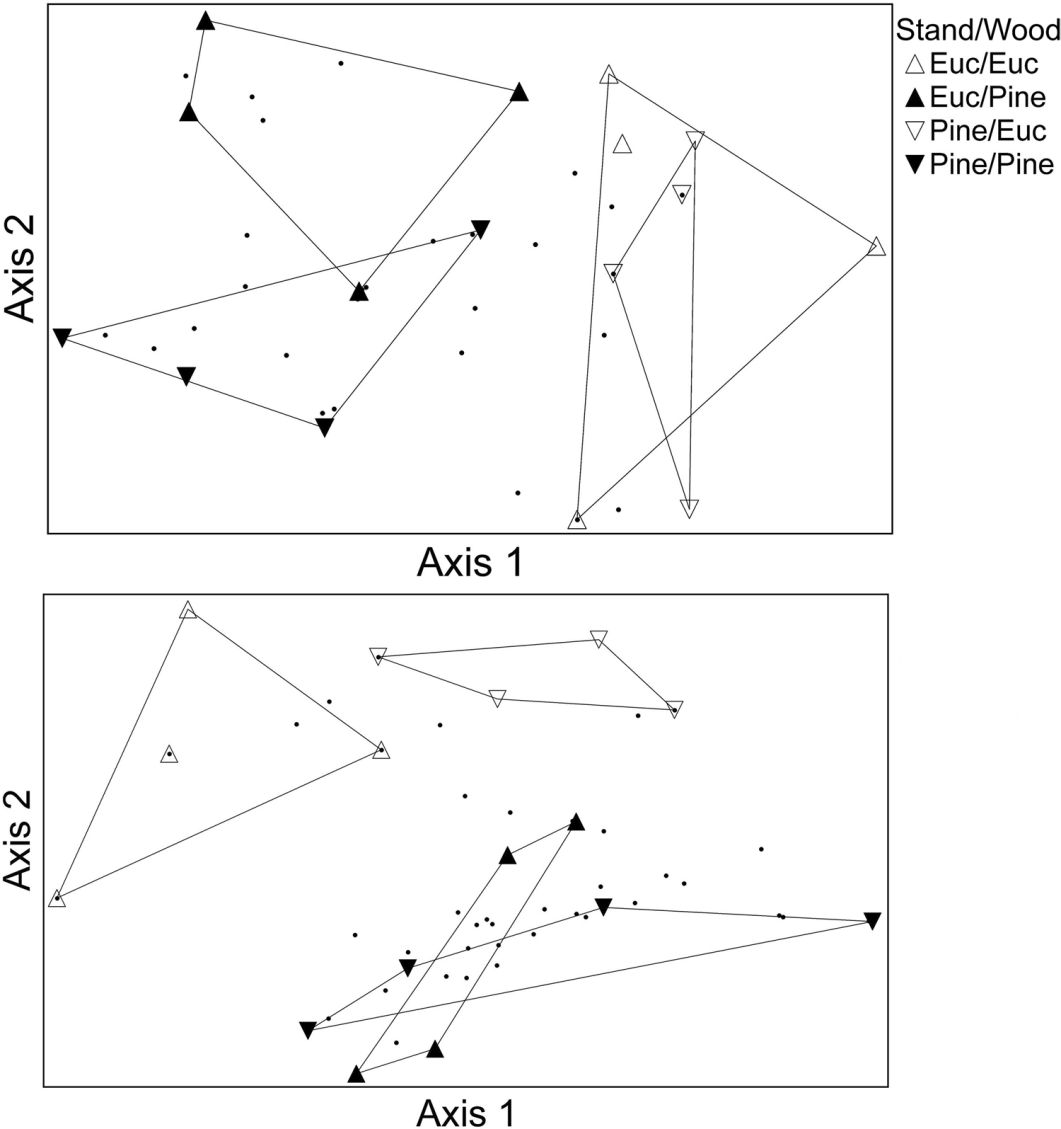
NMDS ordination for beetle communities from 2 month- (top) and 12 month- (bottom) old logs.

**Figure 4:**
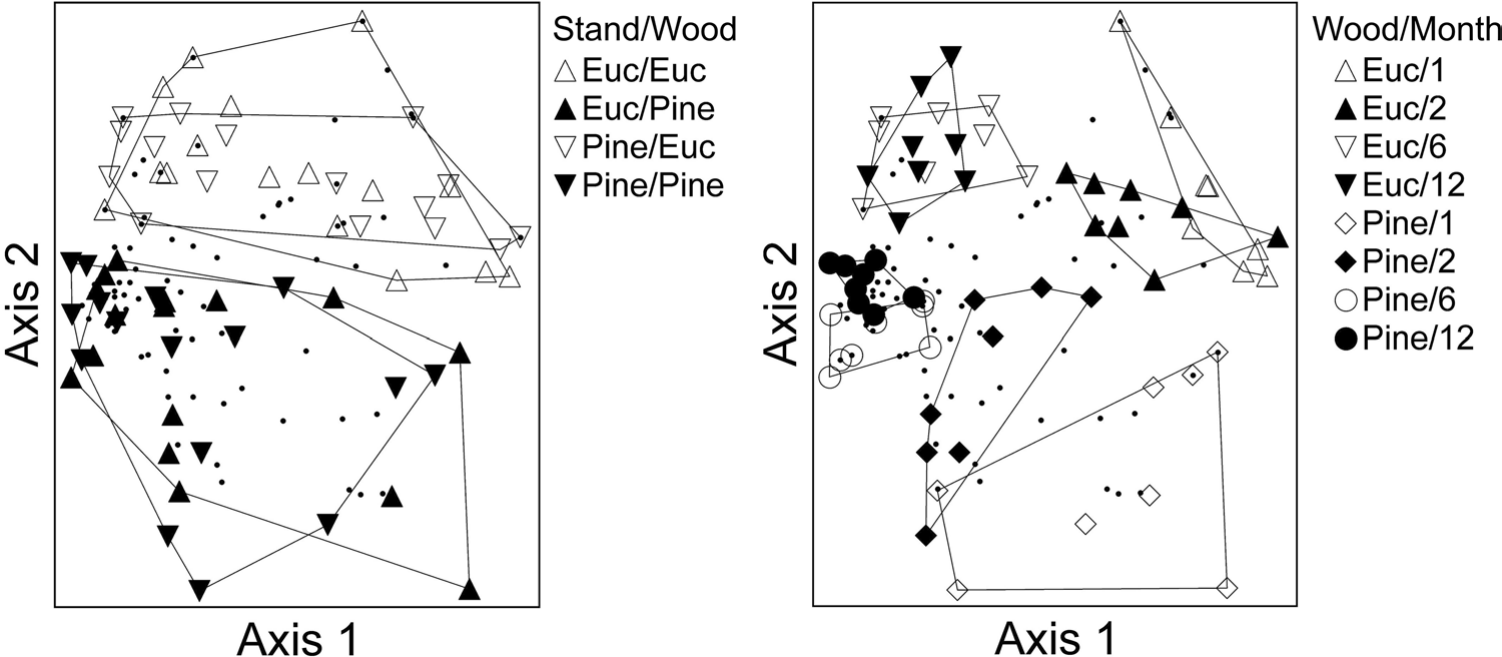
NMDS ordination for beetle communities emerging from logs for all months combined. Samples are grouped by stand type and wood species (left panel) and by wood species and month (right panel).

When analyzing data from each sampling period separately, NMDS and PERMANOVA revealed significant differences in beetle communities between wood species for all sampling periods (Table 3). Only for the 2-month sample did beetle communities differ between stand types (Table 3, Fig. 3). However, there was a significant interaction between stand and wood species for the 12-month sample (Table 3, Fig. 3). When all sampling periods were combined, NMDS and PERMANOVA revealed significant differences in beetle communities between both stand and wood species and there was a significant stand × wood interaction (Table 3, Fig. 4). Based on the same combined dataset, NMDS and PERMANOVA also revealed differences in beetle communities between wood species (F_1,56_ = 6.93, p < 0.001), among months (F_3,56_ = 4.46, p < 0.001), and there was a significant wood × month interaction (F_3,56_ = 2.27, p < 0.001) (Fig. 4).

**Table 3:** Results of two-way PERMANOVA showing effects of stand type, wood species, and their interaction on the composition of beetles emerging from dead logs.

	Stand	Wood	Stand × wood
1 month	F_1,12_ = 0.62, p = 0.89	F_1,12_ = 2.32, p < 0.01	F_1,12_ = 0.75, p = 0.73
2 months	F_1,12_ = 1.89, p = 0.03	F_1,12_ = 3.07, p < 0.01	F_1,12_ = 1.29, p = 0.20
6 months	F_1,12_ = 1.37, p = 0.21	F_1,12_ = 6.53, p < 0.001	F_1,12_ = 1.23, p = 0.26
12 months	F_1,12_ = 1.39, p = 0.15	F_1,12_ = 3.35, p < 0.01	F_1,12_ = 1.86, p = 0.04
All months	F_1,60_ = 1.71, p = 0.03	F_1,60_ = 5.76, p < 0.001	F_1,60_ = 1.61, p = 0.04

## Discussion

Non-native plants have been implicated as a contributing factor to the global decline of insect populations, particularly when they replace native plant species^32^. *Eucalyptus* species have been evaluated for their potential as a forest crop in the U.S. for decades^33^, but little effort has gone towards evaluating potential environmental impacts of this non-native crop tree. This study represents the first effort to assess the value of eucalypt wood to saproxylic insects in the U.S., and one of few such studies globally^5^. Although *Eucalyptus* supports significantly fewer beetle and true bug species than loblolly pine—the most commercially important native timber species in the southern U.S.—we still captured dozens of native species from this novel substrate. However, most of the species collected from *Eucalyptus* were mycophages and predators (Table 1) which are inherently less host specific than phloeoxylophages, a greater diversity of which emerged from native loblolly pine (21 vs. 5 species).

Despite this general pattern, several common native phloeoxylophagous species with broad host ranges were captured from eucalypt wood, including the cerambycid *Knulliana cincta* (Drury), a species reported previously from *Quercus*, *Carya*, *Salix*, and *Ostrya*^34^, as well as many bark beetles belonging to the genus *Hypothenemus*. Not surprisingly given their phylogenetic dissimilarity, eucalypt and pine hosted compositionally distinct insect communities, with several species being found only on eucalypts (e.g., *Monarthrum mali* (Fitch) and *K. cincta*) or pine (e.g., *Asemum striatum* (L.), *Astylopsis sexguttata* (Say), and *Monochamus carolinensis* (Olivier)) (Table 1). This was true for every sampling period although, as predicted, the separation between species appeared to decline over time (Fig. 4).

We recorded five non-native ambrosia beetle species from eucalypt logs in this study compared to two species from pine. This suggests that eucalypts may have a facilitative effect on non-native ambrosia beetles in the region. Over 60 species of non-native bark and ambrosia beetles are established in the United States^35,36^, and typically account for a large proportion of ambrosia beetle species and individuals captured in the southeastern U.S.^37,38^. Previous work on the Savannah River Site, where the current study was conducted, found non-native ambrosia beetles species to account for half the species and up to 86% of individuals collected^39,40^. While the ecological implications of many of these species remain poorly understood, these species do not appear to significantly affect the diversity of native bark and ambrosia beetles^41^.

Contrary to Fierro et al.^5^, we found no evidence that eucalypt litter reduces the richness or diversity of saproxylic insects in logs compared to logs placed on native pine litter. In fact, the opposite pattern was observed for insect diversity (Table 2, Fig. 2). It is not clear if this difference is due to differences in leaf litter chemistry or to some variable unrelated to litter that also differed between stands. For example, logs placed in eucalypt stands experienced more sun exposure than those placed in pine stands because eucalypt stands experienced more mortality than pine (due to both windthrow and frost) and maintained less continuous canopies^23^. Moreover, previous studies have shown a positive correlation between sun exposure and beetle diversity in dead wood, possibly because sun exposure enhances microclimatic heterogeneity within and between dead logs^42,43^. Whatever the explanation, our results provide no support for the idea that eucalypt litter reduces the diversity of saproxylic insects associated with dead wood on the forest floor. We suspect the finding reported by Fierro et al.^5^ may be more related to differences in wood age between treatments than to the effects of eucalypt litter.

## Conclusions

Previous comparisons of saproxylic insect diversity between non-native and native wood species suggest that suitability varies greatly among species and that being non-native to a region does not guarantee that a species will be of little value to saproxylic insects. Indeed, multiple studies have shown that the diversity and composition of insects associated with certain non-native wood species can be comparable to assemblages supported by native wood species^7–10^. While our findings suggest that many insects species native to the southeastern United States can utilize dead *Eucalyptus* wood, this taxon supports a much lower diversity of saproxylic insects than loblolly pine, the native timber species most likely to be displaced by *Eucalyptus* plantations in the region. Such findings are consistent with past research showing that, compared to native forests, non-native tree plantations have a negative effect on numerous taxa^16–18^. The current study provides further support for the conclusion that that plantations consisting of native tree species are more beneficial for biodiversity than those consisting of non-native species^3^.

## Data Availability

The dataset generated during the current study are available from the corresponding author on reasonable request.

## References

[1] FAO (2020). Global Forest Resources Assessment 2020—Key findings.

[2] Brockerhoff EG, Jactel H, Parrotta JA, Quine CP, Sayer J (2008). Plantation forests and biodiversity: Oxymoron or opportunity?. Biodivers. Conserv..

[3] Bremer LL, Farley KA (2010). Does plantation forestry restore biodiversity or create green deserts? A synthesis of the effects of land-use transitions on plant species richness. Biodivers. Conserv..

[4] Ulyshen MD (2018). Saproxylic Insects: Diversity, Ecology and Conservation.

[5] Fierro A, Grez AA, Vergara PM, Ramírez-Hernández A, Micó E (2017). How does the replacement of native forest by exotic forest plantations affect the diversity, abundance and trophic structure of saproxylic beetle assemblages?. For. Ecol. Manag..

[6] Kärvemo S, Schroeder M, Ranius T (2023). Beetle diversity in dead wood is lower in non-native than native tree species, especially those more distantly related to native species. J. Appl. Ecol..

[7] Ulyshen MD, Ulyshen MD (2018). Utilization of non-native wood by saproxylic insects. Saproxylic Insects: Diversity, Ecology, and Conservation.

[8] Vogel S (2021). Diversity and conservation of saproxylic beetles in 42 European tree species: An experimental approach using early successional stages of branches. Insect Conserv. Divers..

[9] Bertheau C (2009). Colonisation of native and exotic conifers by indigenous bark beetles (Coleoptera: Scolytinae) in France. For. Ecol. Manag..

[10] Della Rocca F, Stefanelli S, Bogliani G (2016). *Robinia pseudoacacia* as a surrogate for native tree species for saproxylic beetles inhabiting the riparian mixed forests of northern Italy. Agric. For. Entomol..

[11] Bouget C, Brin A, Larrieu L (2021). The use of sentinel logs to assess host shifts in early beetle colonisers of deadwood under climate- and forestry-induced tree species substitutions. Insect Conserv. Divers..

[12] Ratsirarson H, Robertson HG, Picker MD, Noort SV (2002). Indigenous forests versus exotic eucalypt and pine plantations: A comparison of leaf-litter invertebrate communities. Afr. Entomol..

[13] Wang J, Liao Q-S, Ding W-M, Tong X-L (2008). Invertebrate biodiversity in litter layers of natural forest and Eucalyptus plantation in eastern Guangdong, China. Ying Yong Sheng Tai Xue Bao.

[14] Cifuentes-Croquevielle C, Stanton DE, Armesto JJ (2020). Soil invertebrate diversity loss and functional changes in temperate forest soils replaced by exotic pine plantations. Sci. Rep..

[15] Brockerhoff EG (2013). Role of eucalypt and other planted forests in biodiversity conservation and the provision of biodiversity-related ecosystem services. For. Ecol. Manag..

[16] Goded S (2019). Effects of eucalyptus plantations on avian and herb species richness and composition in North-West Spain. Glob. Ecol. Conserv..

[17] Graça MAS, Pozo J, Canhoto C, Elosegi A (2002). Effects of Eucalyptus plantations on detritus, decomposers, and detritivores in streams. Sci. World J..

[18] Leão-Gomes B, Vasconcelos HL (2023). Land-use changes in a neotropical biodiversity hotspot and its effects on Euglossini bees. J. Insect Conserv..

[19] Ferreira JVA (2022). Critical role of native forest and savannah habitats in retaining neotropical pollinator diversity in highly mechanized agricultural landscapes. Agric. Ecosyst. Environ..

[20] Grove SJ, Forster L (2011). A decade of change in the saproxylic beetle fauna of eucalypt logs in the Warra long-term log-decay experiment, Tasmania. 1. Description of the fauna and seasonality patterns. Biodivers. Conserv..

[21] Grove SJ, Forster L (2011). A decade of change in the saproxylic beetle fauna of eucalypt logs in the Warra long-term log-decay experiment, Tasmania. 2. Log-size effects, succession, and the functional significance of rare species. Biodivers. Conserv..

[22] Gonzalez R (2011). Exploring the potential of Eucalyptus for energy production in the Southern United States: Financial analysis of delivered biomass. Part I. Biomass Bioenergy.

[23] Younger SE, Jackson CR, Dix MJ, Caldwell PV, Aubrey DP (2023). Evapotranspiration partitioning of *Eucalyptus benthamii* and *Pinus taeda* during early stand development. BioEnergy Res..

[24] Ferreira GWD, Rau BM, Aubrey DP (2020). Herbicide, fertilization, and planting density effects on intensively managed loblolly pine early stand development. For. Ecol. Manag..

[25] Ulyshen MD, Hanula JL (2009). Habitat associations of saproxylic beetles in the southeastern United States: A comparison of forest types, tree species and wood postures. For. Ecol. Manag..

[26] Arnett RH, Thomas MC (2000). American Beetles: Vol 1. Archostemata, Myxophaga, Adephaga, Polyphaga: Staphyliniformia.

[27] Arnett RH, Thomas MC, Skelley PE, Frank JH (2002). American Beetles: Vol 2. Polyphaga: Scarabaeoidea through Curculionoidea.

[28] Thomas M (1993). The Flat Bark Beetles of Florida (Coleoptera: Silvanidae, Passandridae, and Laemophloeidae). Arthropods of Florida and Neighboring Land.

[29] Stephan, K. H., Division of Plant Industry, Florida Dept. of Agriculture & Services, C. *The Bothrideridae and Colydiidae of America north of Mexico (Coleoptera: Clavicornia and Heteromera)* (1989).

[30] R Core Team. *R: A Language and Environment for Statistical Computing* (R Foundation for Statistical Computing, 2022).

[31] McCune B, Mefford MJ (2011). PC-ORD. Multivariate Analysis of Ecological Data, Version 6.

[32] Tallamy DW, Narango DL, Mitchell AB (2021). Do non-native plants contribute to insect declines?. Ecol. Entomol..

[33] Kellison RC, Lea R, Marsh P (2013). Introduction of *Eucalyptus* spp. into the United States with special emphasis on the Southern United States. Int. J. For. Res..

[34] Lingafelter SW (2007). Illustrated Key to the Longhorned Woodboring Beetles of the Eastern United States.

[35] Haack R, Rabaglia R (2013). Exotic Bark and Ambrosia Beetles in the USA: Potential and Current Invaders.

[36] Gomez DF, Rabaglia RJ, Fairbanks KEO, Hulcr J (2018). North American Xyleborini north of Mexico: A review and key to genera and species (Coleoptera, Curculionidae, Scolytinae). ZooKeys..

[37] Hartshorn JA, Coyle DR, Rabaglia RJ (2021). Responses of native and non-native bark and ambrosia beetles (Coleoptera: Curculionidae: Scolytinae) to different chemical attractants: Insights from the USDA Forest Service early detection and rapid response program data analysis. J. Econ. Entomol..

[38] Sheehan TN, Ulyshen MD, Horn S, Hoebeke ER (2019). Vertical and horizontal distribution of bark and woodboring beetles by feeding guild: Is there an optimal trap location for detection?. J. Pest Sci..

[39] Coyle DR, Brissey CL, Gandhi KJK (2015). Species characterization and responses of subcortical insects to trap-logs and ethanol in a hardwood biomass plantation. Agric. For. Entomol..

[40] Ulyshen MD, Sheehan TN (2019). Trap height considerations for detecting two economically important forest beetle guilds in southeastern US forests. J. Pest Sci..

[41] Hartshorn JA, Coyle DR (2021). Comparative meta-analysis effects of nonnative ants (Hymenoptera: Formicidae), ground beetles (Coleoptera: Carabidae), and bark and ambrosia beetles (Coleoptera: Curculionidae) on native confamilials. Environ. Entomol..

[42] Lindhe A, Lindelöw Å, Åsenblad N (2005). Saproxylic beetles in standing dead wood density in relation to substrate sun-exposure and diameter. Biodivers. Conserv..

[43] Lettenmaier L (2022). Beetle diversity is higher in sunny forests due to higher microclimatic heterogeneity in deadwood. Oecologia.

